# Role of season, temperature and humidity on the incidence of epistaxis in Alberta, Canada

**DOI:** 10.1186/1916-0216-43-10

**Published:** 2014-04-22

**Authors:** Leigh J Sowerby, Joshua J DeSerres, Luke Rudmik, Erin D Wright

**Affiliations:** 1 Department of Otolaryngology - Head and Neck Surgery, Western University, London, ON, Canada; 2 Faculty of Medicine and Dentistry, University of Alberta, Edmonton, AB, Canada; 3 Division of Otolaryngology – Head & Neck Surgery, University of Calgary, Calgary, AB, Canada; 4 Division of Otolaryngology-Head & Neck Surgery, University of Alberta, Edmonton, AB, Canada; 5 Alberta Sinus Centre, University of Alberta Hospital, Room 1E4 WMC, 8440 – 112 Street, Edmonton, AB T6G 2B7, Canada

**Keywords:** Epistaxis, Season, Temperature, Humidity, Admission, Age

## Abstract

**Background:**

Classical dogma holds that epistaxis is more common in winter months but there is significant variability reported in the literature. No study has yet examined the effect of season, humidity and temperature on epistaxis in a location with as severe weather extremes as seen in Alberta, Canada. The objective of the study is to evaluate for an effect of these meteorological factors on the incidence of epistaxis in Alberta.

**Method:**

A retrospective review of consecutive adult patients presenting to the Emergency room (ER) in Edmonton and Calgary, Alberta over a three-year period was performed. Daily temperature and humidity data was recorded from the respective airports. Statistical analysis with Pearson’s correlation coefficient was performed.

**Results:**

4315 patients presented during the study period. Mean daily temperatures ranged from a low of -40°C to a high of +23°C. A significant negative correlation was found for mean monthly temperature with epistaxis (Pearson’s r = -0.835, p = 0.001). A significant correlation was also present for daily temperature and epistaxis presentation (Pearson’s r = -0.55, p = 0.018, range 1.8 to 2.2 events/day). No correlation was identified with humidity and no significant seasonal variation was present.

**Conclusions:**

A negative correlation was found to exist for both daily and mean monthly temperature with rates of epistaxis. A seasonal variation was seen in Edmonton but not in Calgary. No correlation was found for humidity when compared to both presentation rates and admissions.

## Background

In medical school, students are taught that epistaxis is more common in winter months. Several studies have examined the relationship between season, temperature, humidity and the presentation of epistaxis. The majority of these studies have shown a correlation with case frequency and at least one of the above-mentioned meteorological factors, [1-8] but other studies have shown no relationship [9-11]. Despite numerous reports on this topic, no known studies have been performed in a location with temperature fluctuations as great as in Alberta, Canada. The purpose of this study is to examine whether a correlation exists between season, temperature and humidity and the rate of epistaxis presentation to the Emergency room (ER) in adult patients.

## Methods

A retrospective review of adult ER visits was performed from January 2008 to December 2010 in Edmonton, Alberta and Calgary, Alberta, Canada. This review was approved by the respective Health Research Ethics Boards (Edmonton study ID Pro00025159 and Calgary Study ID 24207). In Edmonton, the study included the two major tertiary care hospitals (the Royal Alexandra Hospital (RAH) and the University of Alberta Hospital (UAH)); in Calgary, the review included all adult hospitals (the Peter Lougheed Centre, the Rockyview Hospital and the Foothills Medical Centre) within city limits. All encounters with an ICD-9 (International Classification of Disease, Ninth Revision) diagnostic code for epistaxis (784.7) were reviewed for more detailed analysis. Patients presenting with epistaxis as a primary diagnosis, including patients with spontaneous bleeds and patients with bleeding secondary to medical co-morbidities, were included in the study. To truly tease out a weather effect on epistaxis, patients with epistaxis secondary to trauma were excluded, and errors in the ICD-9 coding of patients were excluded from analysis as well. Patients presenting within 2 weeks of the initial epistaxis event with another episode of epistaxis were considered to be the same epistaxis event; patients presenting with another epistaxis after 2 weeks were considered to be a new epistaxis event.

Mean daily temperature and humidity readings from both the Calgary and Edmonton airport were collected from Environment Canada [12] for the same time period. Seasons were defined as follows: Winter included December, January and February; Spring included March, April and May; Summer included June, July and August; and, Fall included September, October and November.

All inferential tests were two-tailed and p = 0.05 was set as the threshold for statistical significance. Post-Hoc Tukey HSD (honestly significant difference) testing was used to test for significance between groups and Chi-squared testing was used to compare ordinal variables. An *a priori* decision was made to consider correlation coefficients, r, between -0.4 and +0.4 inclusive weak correlations, between 0.4 and 0.7 or between -0.4 and -0.7 moderate correlations; and ≥ 0.7 or ≤ -0.7 strong correlations. All analyses were performed using the statistical software package SPSS version 20.0 (Chicago, Illinois).

## Results

### Case presentations

**Figure 1:**
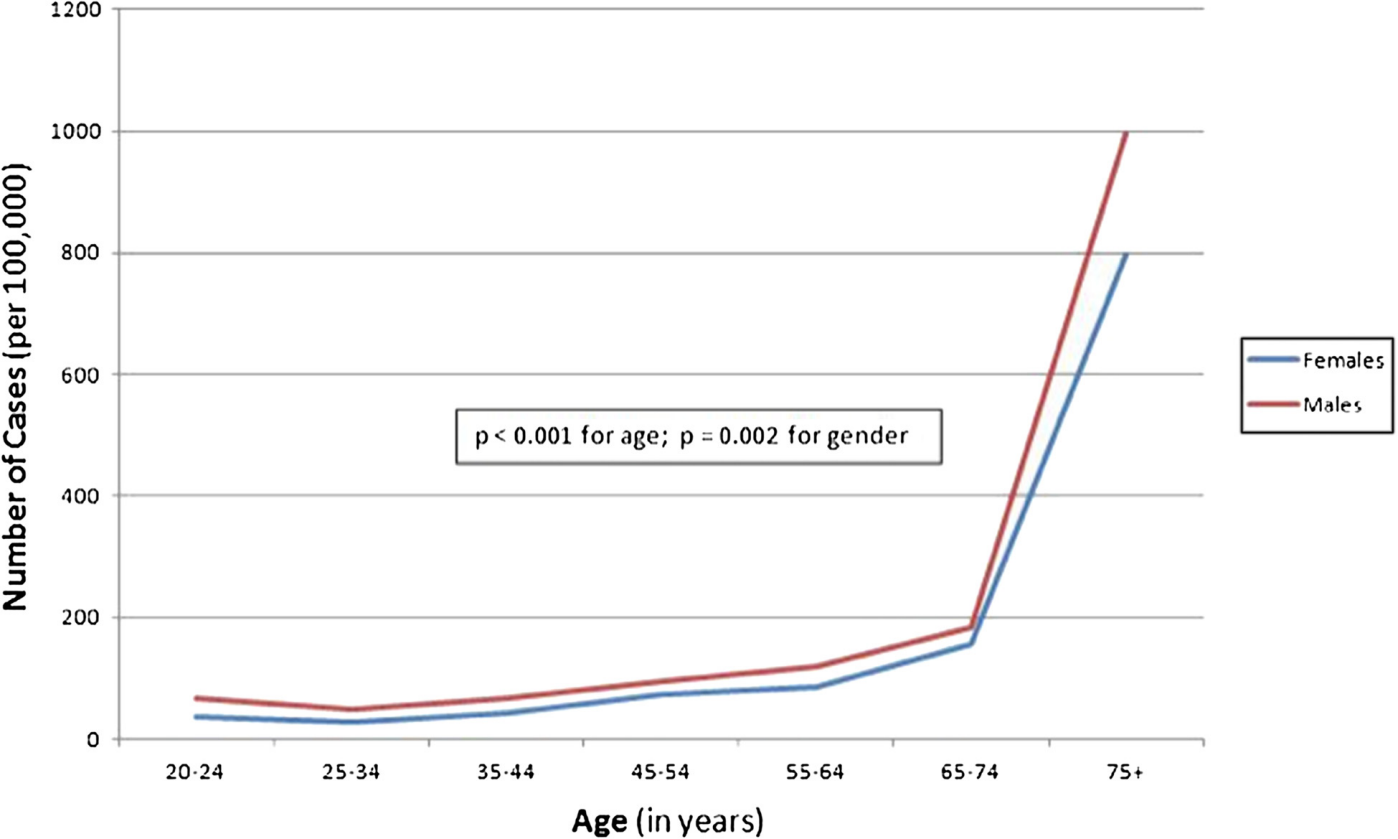
Incidence of epistaxis cases presenting to hospital in Calgary in 2009 according to age.

A total of 4315 cases of epistaxis were identified, with 2770 discrete events in Calgary and 1545 events in Edmonton (Table 1). There was a visually clear association between age and epistaxis incidence, with the number of cases rising with age, even though overall population numbers tended to do the reverse (Figure 1). Males (56.2%) also outnumbered females, both in absolute numbers and in incidence in every age group (Figure 1).

**Table 1:** Epistaxis case presentations in Calgary and Edmonton from 2008-2010

	**Calgary**	**Edmonton**	**Overall**
**Cases in 2008**	934	537	1471
**2009**	933	543	1476
**2010**	903	464	1367
**Total cases**	2770	1545	4315
**Average age of patients (years)**	63.7	60.9	62.7

The smaller communities in Edmonton are also served by community hospitals for which epistaxis data was not available. As such, age and gender-adjusted incidence were calculated for Calgary alone. Census data by both age and gender was only available for 2009; as such, the incidence data for 2009 is presented in Table 2. As demonstrated in Table 2, the incidence of epistaxis in Calgary was slightly higher for men than women (122.8 per 100,000 vs 106.9 per 100,000).

**Table 2:** Incidence of epistaxis by age and sex in Calgary in 2009 (per 100,000)

**Age-adjusted incidence in Calgary - females - 2009**			
**Age**	**# cases**	**Population**	**Incidence**
20-24	14	39,429	35.5
25-34	25	90,302	27.7
35-44	38	87,279	43.5
45-54	59	81,763	72.2
55-64	42	49,416	85.0
65-74	45	28,780	156.4
75+	208	26,138	795.8
**Total**	**431**	**403,107**	**106.9**
**Age-adjusted incidence in Calgary - males - 2009**			
**Age**	**# cases**	**Population**	**Incidence**
20-24	28	41,302	67.8
25-34	44	93,089	47.3
35-44	63	92,486	68.1
45-54	80	85,655	93.4
55-64	61	51,118	119.3
65-74	48	25,976	184.8
75+	176	17,678	995.6
**Total**	**500**	**407,304**	**122.8**
**Total yearly incidence (per 100,000) =**			**114.9**

**Figure 2:**
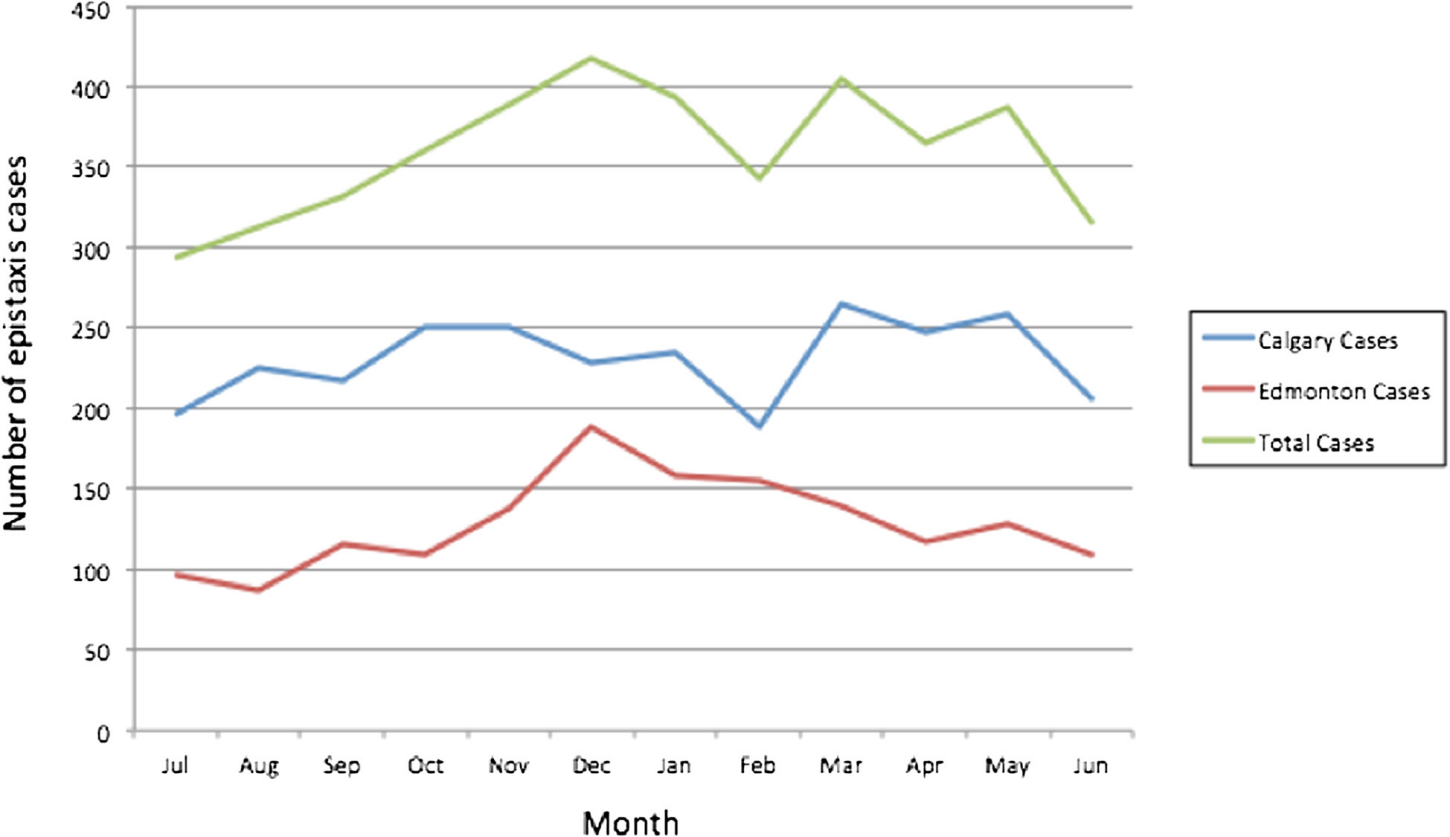
Number of epistaxis cases by month in Calgary and Edmonton from 2008–2010.

**Figure 3:**
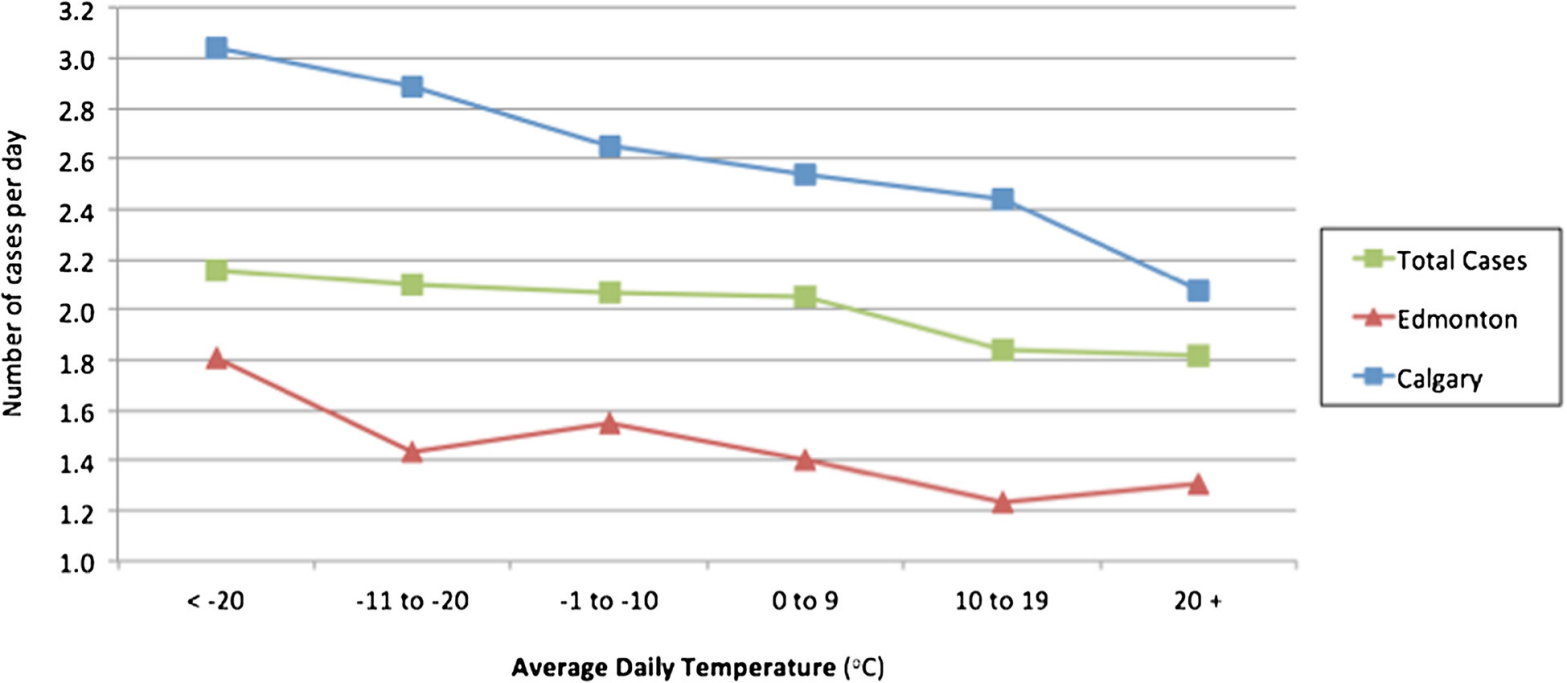
Number of epistaxis cases per day as a function of temperature in Calgary and Edmonton from 2008–2010.

**Figure 4:**
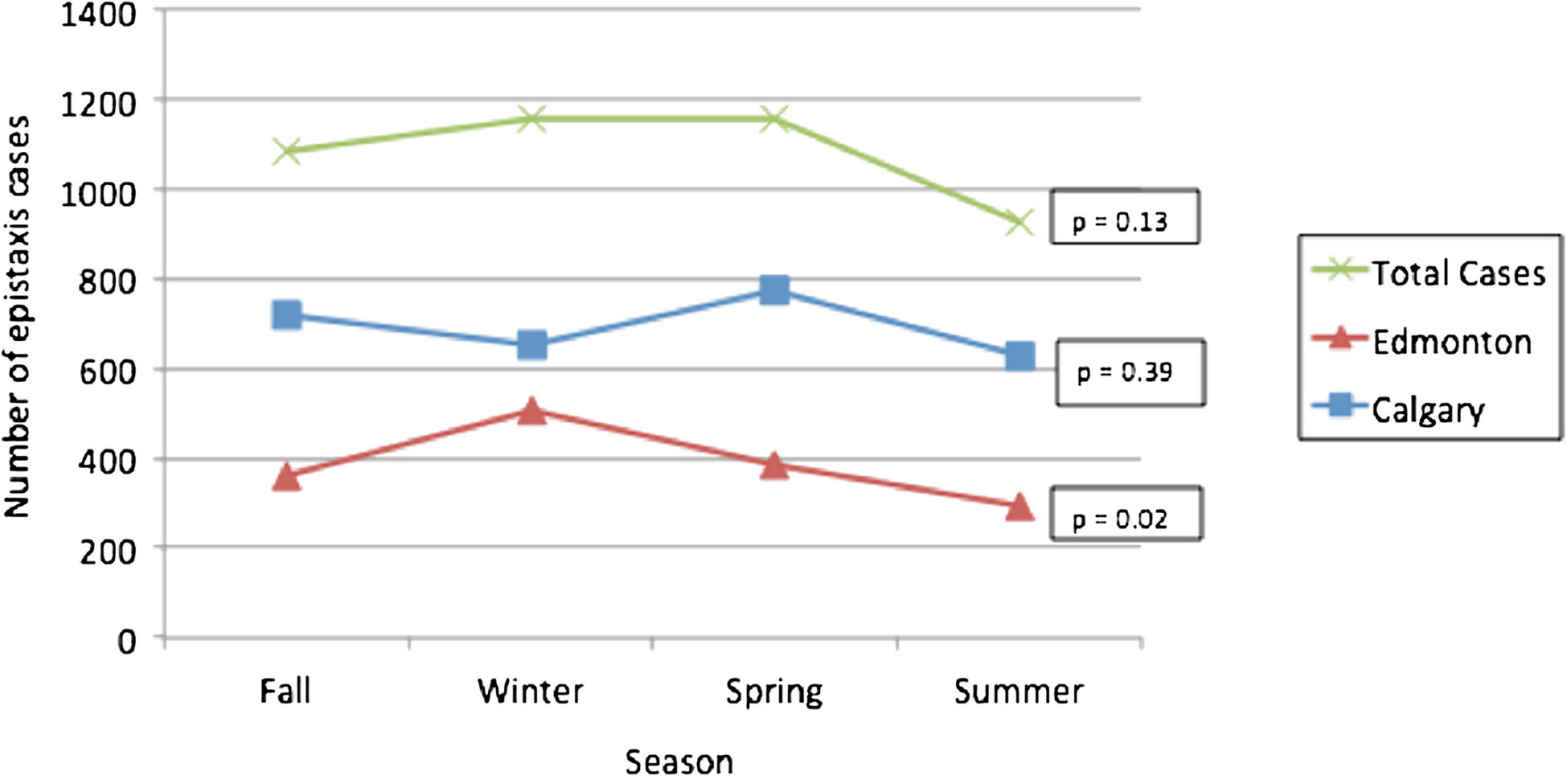
Number of epistaxis cases by season in Calgary and Edmonton from 2008–2010.

**Figure 5:**
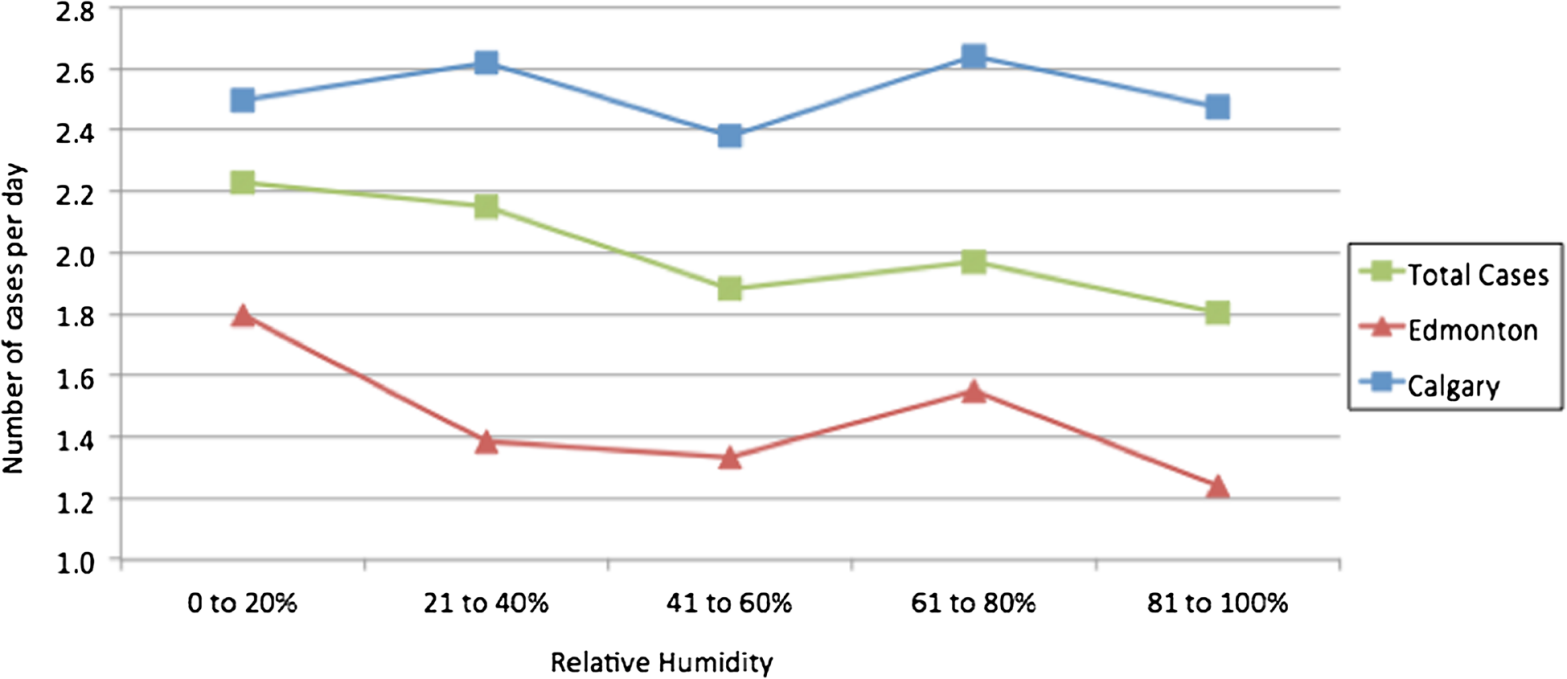
Number of epistaxis cases per day as a function of humidity in Calgary and Edmonton from 2008–2010.

Neither temperature nor humidity varied significantly from year to year over the three years of observation (2008 through 2010) in Calgary and Edmonton. Monthly rankings for humidity and temperature were correlated between Calgary and Edmonton and temperature ranking was correlated with humidity ranking in Edmonton (r = 0.71, p = 0.010), but not in Calgary (r = 0.46, p = 0.13). A strong negative correlation was found to exist between temperature and epistaxis case frequency when temperature was evaluated by ranking months from coldest to warmest (Figure 2). This is true in Calgary (r = -0.80, r2 = 0.64, p = 0.002); Edmonton (r = -0.77, r2 = 0.0.59, p = 0.004); and overall (r = -0.835, r2 = 0.70, p = 0.001). As depicted in Figure 2, there was a drop in cases of approximately 30% from the coldest month to warmest (417 cases in December to 293 cases in July). When mean daily temperature was examined, a significant inverse relationship was seen in both cities (Calgary - r = -0.99; p < 0.001, Edmonton - r = -0.81; p = 0.049) and overall (r = -0.55; p = 0.018) (Figure 3). The number of total cases per day dropped from 2.2 when the temperature was below minus 20°C to 1.8 when the temperature was greater than 20°C. A significant difference was seen between seasons in Edmonton (p = 0.02), but not in Calgary (p = 0.39), or in overall cases (p = 0.13) (Figure 4). In absolute numbers, over the three years, 1154 cases (26.7%) occurred in the winter while 923 cases (21.4%) were in summer months. When mean daily relative humidity was examined, no significant relationship was noted with cases of epistaxis (Figure 5). This also held true when evaluating humidity by ranking months from least to most humid.

### Hospital admissions

**Figure 6:**
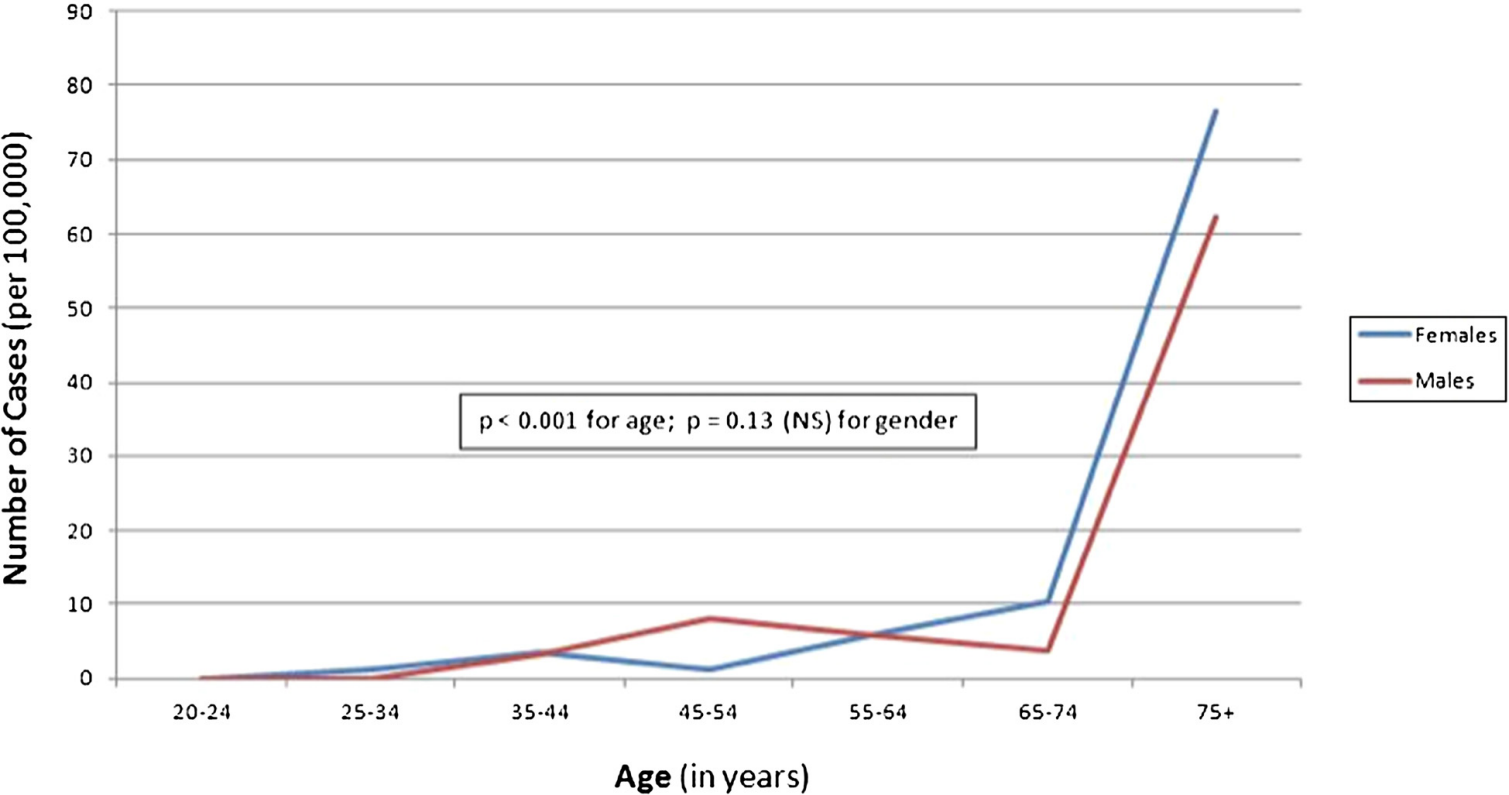
Incidence of epistaxis requiring admission to hospital in Calgary in 2009 according to Age.

When only patients admitted to hospital were considered, 337 cases (7.8% of all cases presenting to ER) were admitted over the three-year period. Interestingly, there were more patients admitted with epistaxis in Edmonton than in Calgary, conflicting with the lower number of overall cases of epistaxis seen. The admission rate in Edmonton (173 of 1545, 11.2%) was almost double the rate in Calgary (164 of 2770, 5.9%), and this difference was statistically significant (*χ*2 = 38.36, df = 1, p < 0.001). In 2009, the incidence of admissions for adults in Calgary, by age and gender, was 7.7 per 100,000 adult females and 6.1 per 100,000 adult males, for an overall incidence of 6.9 per 100,000. In general, hospitalizations rose with age, but less linearly than with overall cases. Again, as with all epistaxis cases, there was a precipitous rise in hospital admissions beginning at age 75 and above (Figure 6).

**Figure 7:**
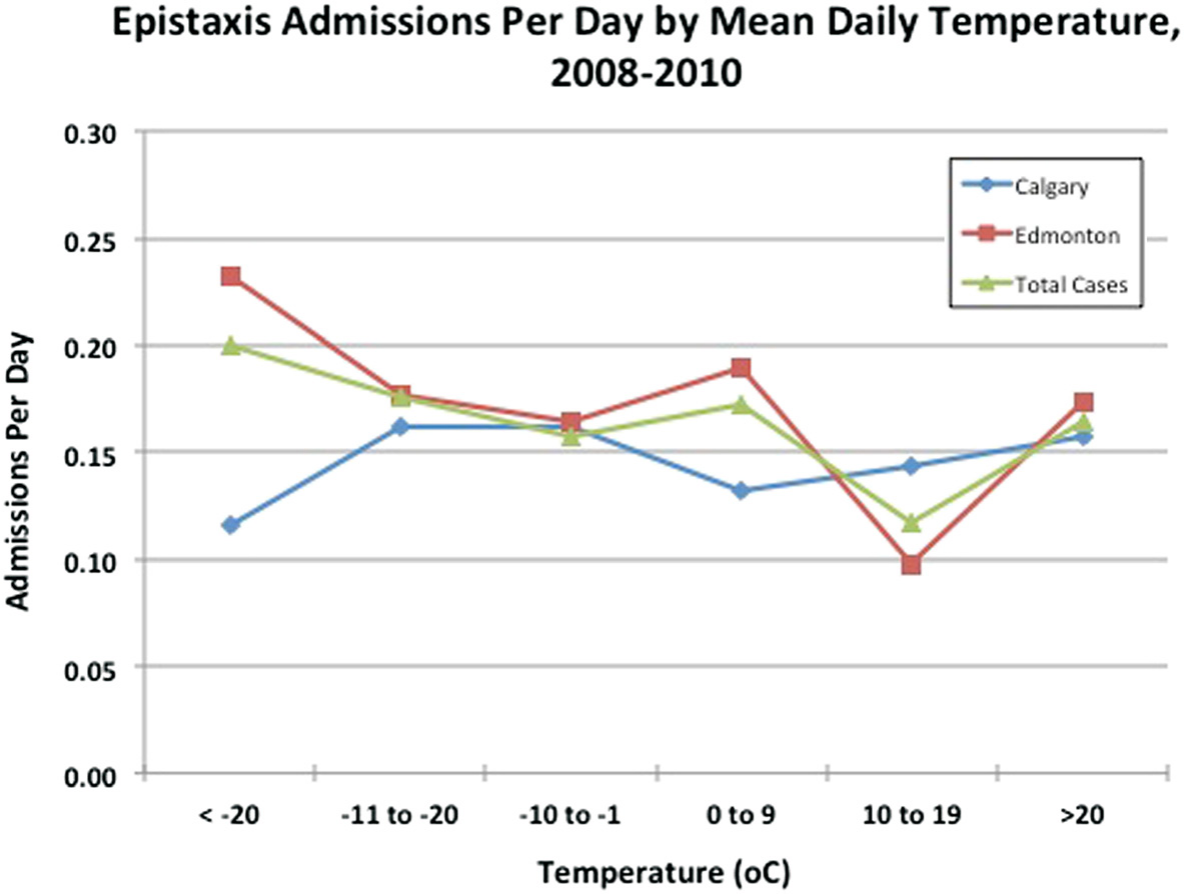
Epistaxis admissions per day as a function of mean daily temperature, in Calgary and Edmonton from 2008–2010.

**Figure 8:**
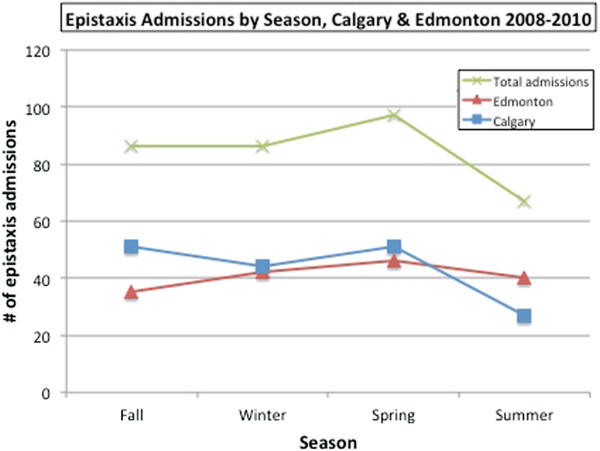
Epistaxis admissions as a function of season in Calgary and Edmonton from 2008–2010.

As with all cases of epistaxis (admitted and not admitted), males (57.9%) outnumbered females overall. The relationship between average monthly temperature and admission rate only approached significance (*χ*2 = -0.533, p = 0.075), and it was lost altogether when Edmonton (*χ*2 = -0.488, p = 0.11) and Calgary (*χ*2 = -0.222, p = 0.49) were analyzed alone. As with the entire sample of 4315 cases, month rank by humidity did not correlate with the number of admissions in either city, or overall. When admissions were examined by mean daily temperature, a significant inverse relationship was seen in Edmonton (r = -0.57; p = 0.04), but not in Calgary (r = 0.34, p = 0.26) nor overall (r = -0.30; p = 0.32) (Figure 7). The number of admissions per day dropped from 0.23 in Edmonton when the temperature was below minus 20°C to 0.17 when the temperature was greater than 20°C. Interestingly, a significant difference was seen in seasonal admissions in Calgary (p = 0.03), but not in Edmonton (p = 0.67), nor in overall cases (p = 0.14) (Figure 8). The average number of admissions per season in Calgary was 43, with a decrease to 27 in the summer months.

## Discussion

This retrospective study sought to examine the relationship between the incidence of epistaxis and season, temperature and humidity. It is the first study to investigate these relationships in a climate with significant seasonal weather extremes. This study has demonstrated that there is, indeed, an inverse relationship between mean daily temperature and epistaxis presentation. When evaluating the correlation between season and epistaxis presentation, a significant correlation was only found for Edmonton, while a seasonal variation in admission rates was only present for Calgary. In both instances, it was a decrease in cases in summer months (as compared to all other months of the year which demonstrated similar rates) rather than the traditionally taught increase in winter months. When months were ranked from coldest to warmest, a significant negative correlation was found for case presentation, with a peak incidence in colder months. When the daily epistaxis presentation was compared to mean daily humidity, no correlation was found for either Calgary, Edmonton, or with overall cases. A statistically significant higher rate of admission was found for Edmonton compared to Calgary.

Possible explanations for the relationship between case presentations according to season and temperature include increased use of forced air heating and potential increase in the frequency of URTIs (upper respiratory tract infections) during colder times [13]. Some studies have also shown a decrease in coagulation in colder temperatures [14]. Although unseasonably high and low temperatures could cause variability in presentation rates, average monthly temperatures remained fairly consistent within each city over the course of the three years included in this study (Table 3).

**Table 3:** Average monthly temperatures in degrees celsius in Calgary and Edmonton from 2008 to 2010

	**2008**		**2009**		**2010**	
	**Calgary**	**Edmonton**	**Calgary**	**Edmonton**	**Calgary**	**Edmonton**
**January**	-8.5	-14.6	-6.2	-13.2	-6.2	-12.1
**February**	-5.2	-11.3	-7.1	-13.7	-7.1	-9.1
**March**	0.0	-3.6	-4.4	-11.0	-4.4	-0.9
**April**	1.5	0.8	3.4	2.2	3.4	5.6
**May**	10.7	11.0	9.7	8.3	9.7	8.1
**June**	13.3	13.9	13.3	12.6	13.3	13.9
**July**	16.5	15.6	17.0	15.5	17.0	15.6
**August**	16.6	16.0	15.9	14.7	15.9	14.4
**September**	11.3	10.1	15.0	13.3	15.0	8.2
**October**	6.7	4.3	1.5	1.1	1.5	5.8
**November**	2.2	-0.2	1.9	-1.0	1.9	-7.0
**December**	-10.9	-16.3	-12.5	-18.8	-12.5	-15.3

As mentioned previously, our data showed a statistically significant difference in admissions between the two cities, with patients in Edmonton more likely to be admitted. This discrepancy could be related to patients seen at tertiary centres – the UAH and RAH – being more likely to be admitted than those at the other ER sites in Edmonton. Another explanation is that potential admissions may have been referred to UAH or RAH. It also suggests, given that Edmonton is a smaller city, the overall incidence of epistaxis admissions in Edmonton might actually be higher than in Calgary.

When examining the demographics of the patients in this study, there were statistically significant differences between the 2 cohorts, both in terms of age and sex. The Calgary cohort was slightly older and had a higher percentage of females. Unfortunately, Edmonton did not include all hospitals, as data for the smaller community hospitals was not available. However, the majority of the city population is served by the two included hospitals (RAH and UAH). The data for Calgary was obtained from all three of the adult hospitals in the city. Note that these estimates may be over-estimates, because some cases of epistaxis may have involved individuals residing outside the Calgary census area.

As this study examined presentations to ER only, a true rate of incidence was unable to be calculated, as primary care facilities were not included. Although there may have been a large number of patients who presented to their primary care provider – who are not accounted for in this study – examining presentation rates at all levels of care was not the purpose of this study. This study served to examine the change in epistaxis presentation rates according to our three variables, and we assume that any change in frequency would present itself in all levels of care.

The reason for admission as well as information regarding definitive therapy for treatment was not available in the database used for the study. In addition, the sample size precluded an individual chart review and therefore cases could have been missed in the database used as cases were identified based on proper ICD-9 coding. However, we would expect the number of cases missed to be similarly distributed throughout the seasons and temperatures but this does allow for potential error in our study.

This is the first study to be completed in a setting with as significant weather variation as seen in Alberta. As previously mentioned, the classical teaching holds that epistaxis is more frequent in winter months. However, although a negative correlation with temperature was found for both cities, a seasonal variation in presentation was only found in Edmonton. As season classification does not change based on latitude, the weather experienced in Alberta may not reflect what is thought of as ‘seasonal weather’. Therefore, our study shows that epistaxis is most accurately correlated with temperature. These findings allow us to question the classical teaching that epistaxis is more common in winter. Since temperature is often closely associated with season, the seasonal correlation may have been a misinterpretation of what is in fact a correlation with temperature. For instance, we can see that in Calgary – a city with significant temperature variation within seasons, due to the presence of Chinooks – although a correlation with temperature is present, a seasonal variation is not. The findings for this study could be representative for regions with a similar climate, but may not be valid in other locations. As was seen with the results of this study, two cities within 300 km from one another can have significantly different results. Since Edmonton is known to have more extreme weather conditions than Calgary, we could hypothesize that meteorological variation may need to be large enough for a statistically significant seasonal variation in epistaxis presentation to be evident. However, it is important to note that our study was a population-based study and only examined the incidence of epistaxis. Therefore, we can only speculate on the causation of the variations that were seen.

Future directions for this study would be to examine epistaxis rates in relation to patterns of change in temperature, such as with Chinooks in Calgary, Alberta. This may help explain why both cities showed a negative correlation with temperature but only Edmonton showed a seasonal variation. Another approach could be to record daily temperatures and humidity in Alberta homes and compare these values to presentation rates, since the majority of time is spent indoors, especially during colder months. The effectiveness of education for intervention could also be examined, such as the use of humidification in homes.

## Conclusions

This study showed a strong negative correlation between temperature and epistaxis presentation for both Calgary, Edmonton and overall but was only statistically significant for admissions in Edmonton. No correlation was found for humidity when evaluating both case presentation and admission rates. A seasonal relationship for epistaxis presentation was found only in Edmonton. A difference in admissions was only present in Calgary, with a decrease in summer months. Overall, there was no significant seasonal relationship with epistaxis. This data supports the notion that epistaxis is more common in colder weather, but the clinical difference in rates of presentation was small.

## Abbreviations

ER: Emergency room; RAH: Royal Alexandra Hospital; UAH: University of Alberta Hospital; ICD-9: International Classification of Disease – Ninth Revision; HSD: Honestly significant difference; URTI: Upper respiratory tract infection.

## Competing interests

The authors declare that they have no competing interests.

## Authors’ contributions

LS conceived the idea and contributed to the design of the study, interpretation of data and drafted the manuscript. JD helped in conception and design of the study, acquisition of data and helped to draft the manuscript. LR contributed to acquisition of data and was involved in critical revision of the manuscript. EW participated in the study design and coordination, and data analysis and was involved in critical revision of the manuscript. All authors read and approved the final manuscript.

## Confirmation of original material

This material has never been published and is not currently under evaluation in any other peer-reviewed publication.

## Meeting presentation

Oral presentation at the annual Canadian Society of Otolaryngology – Head and Neck Surgery in Banff, Alberta. Presented June 2013.
